# Low-Power Photoplethysmogram Acquisition Integrated Circuit with Robust Light Interference Compensation

**DOI:** 10.3390/s16010046

**Published:** 2015-12-31

**Authors:** Jongpal Kim, Jihoon Kim, Hyoungho Ko

**Affiliations:** 1 Samsung Electronics Inc., Suwon 16678, Korea; jongpalk@samsung.com; 2 Chungnam National University, Daejeon 34134, Korea; jihoonkim@cnu.ac.kr

**Keywords:** photoplethysmogram, transimpedance amplifier, ambient light cancellation, automatic offset compensation, automatic emitted light compensation

## Abstract

To overcome light interference, including a large DC offset and ambient light variation, a robust photoplethysmogram (PPG) readout chip is fabricated using a 0.13-μm complementary metal–oxide–semiconductor (CMOS) process. Against the large DC offset, a saturation detection and current feedback circuit is proposed to compensate for an offset current of up to 30 μA. For robustness against optical path variation, an automatic emitted light compensation method is adopted. To prevent ambient light interference, an alternating sampling and charge redistribution technique is also proposed. In the proposed technique, no additional power is consumed, and only three differential switches and one capacitor are required. The PPG readout channel consumes 26.4 μW and has an input referred current noise of 260 pArms.

## 1. Introduction

The photoplethysmogram (PPG) is widely used for monitoring cardiovascular parameters such as heartbeat rate, blood vessel stiffness, and pulse transit time blood pressure [1,2,3,4]. In particular, wearable pulse rate sensors based on the PPG have become increasingly popular because of their convenience compared to electrocardiogram sensors. The operation principle of PPG sensors is optical detection of blood volume changes. The sensor system consists of photodiodes (PDs), red, infrared, or green light-emitting diodes (LEDs), and a transimpedance amplifier (TIA). The PPG sensor monitors changes in the light intensity via reflection from the skin or transmission through the tissue [5].

In a PPG monitoring system, light waves emitted from a known and controllable optical source illuminate the fingertip, wrist, or earlobe, and the resultant transmitted or reflected light waves are detected by a PD. A PPG probe is generally worn on the fingertips because a higher signal amplitude can be achieved there than at other sites. Recently, different measurement sites for PPG sensors have been explored extensively for wearable applications, including the ring finger [6], wrist [7], brachia [8], and earlobe [9].

According to the measurement principle, in addition to the signal component of interest, two main interference components are induced by the ambient light intensity and optical path variation. These interferences can be either a direct current (DC) or an alternating current (AC) component of a detected signal. The DC offset of the PPG waveform corresponds to the detected transmitted or reflected optical signal from the tissue and depends on the structure of the tissue and the average blood volume of both arterial and venous blood. The AC component shows changes in the blood volume that occur between the systolic and diastolic phases of the cardiac cycle [5]. A DC offset that depends on the static ambient light intensity and skin color is present in a detected signal. The DC offset is typically a hundred times larger than the pulsatile amplitude [3]. Signal saturation from the large DC offset can be prevented by using a wide dynamic range design, but this results in inefficient power consumption. Therefore, the DC offset should be removed before the detected signal is amplified. Owing to variation in the ambient light intensity or optical path condition between the light source and the detector, the unwanted AC component is present in the detected signal. An ambient light, such as the sun or a fluorescent lamp, can be a noise source because it is unknown and unpredictable.

In previous works, this ambient light was cancelled by a feedback technique with a digital-to-analog converter (DAC) and correction circuit blocks [3] or by an additional analog-to-digital converter (ADC) and a digital signal processor [10]. Other studies also reported a few methods of reducing interference, such as rejecting the DC offset by a low-frequency feedback loop [11], extending the dynamic range using a logarithmic amplification [12], using digital feedback [13], and using an adaptive digital filter [14,15].

This study presents a low-power PPG readout integrated circuit (IC) that is robust to the DC light offset and time-varying interference [16]. Three compensation methods are adopted in this study: (1) a saturation detection and current compensation method to reduce the large DC current offset, (2) ambient light cancellation (ALC) through alternating sampling and charge redistribution, and (3) automatic emitted light compensation (AEC) against optical path variation.

## 2. Circuit Design

### 2.1. Top Architecture and Overall Work Flow

**Figure 1:**
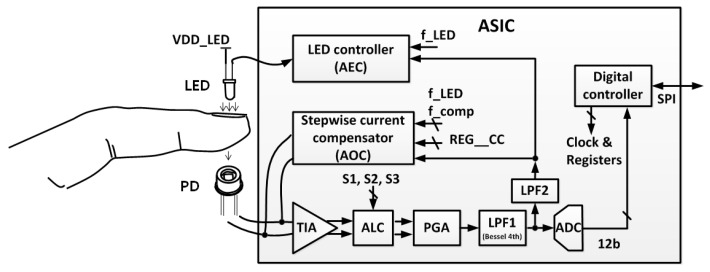
Top block diagram of LED driving and PD detection circuits for PPG application. (TIA: TransImpedance Amplifier; ALC: Ambient Light Cancellation; PGA: Programmable Gain Amplifier; LPF: Low-Pass Filter; PD: PhotoDiode; LED: Light-Emitting Diode; AOC: Automatic Offset Compensation; AEC: Automatic Emitted Light Compensation.)

A top view of the designed architecture is shown in Figure 1. The LED controller controls the amount of light emitted from the LED. The LED is turned on and off for lower power consumption according to the control signal f_LED. The amount of current passing through the LED is controlled by the LED controller depending on the output signal from a low-pass filter (LPF2). The current signal detected by the PD is converted into a voltage signal in the TIA block, and the ambient light effect is cancelled in the ALC block. A considerable amount of the DC offset from the PD is compensated by the current compensator so that it does not saturate a signal at the ADC input stage. The first low-pass filter (LPF1) has a cutoff frequency of 70 Hz to suppress the ADC noise, and the second low-pass filter (LPF2) has a cutoff frequency of 0.1 Hz to extract the DC component.

### 2.2. Fully Differential Transimpedance Amplifier (TIA)

**Figure 2:**
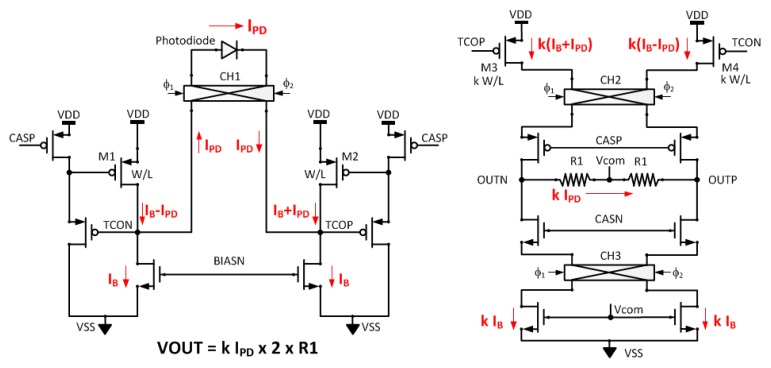
Schematic diagram of fully differential TIA.

A schematic of the fully differential TIA is shown in Figure 2. The PD is operated in photovoltaic mode in a fully differential manner. In photovoltaic mode, a reduced dark current, higher linearity, and lower noise can be obtained than in the photoconductive mode. The fully differential architecture also effectively reduces common mode noises.

To reduce the noise level, combined chopper stabilization and power gating is applied. Chopper stabilization is an effective technique for reducing the low frequency flicker noise components [17]. Flicker noise is observed when the state of the transistor is periodically switched from inversion to accumulation by switching the gate bias [18]. We combine the chopper stabilization and switched bias techniques to reduce both the noise and the power consumption. The gate bias voltages, such as CASP, CASN, and BIASN, are periodically switched, and the bias current of the transistors is switched between operating points and off-states; thus, the average current consumption can be reduced while low noise performance is maintained.

The photocurrent generated from the PD, *I_PD_*, is modulated by the input chopper (CH1). The modulated photocurrent and two bias currents, *I_B_*, form two differential currents, *I_B_ + I_PD_* and *I_B_* − *I_PD_*. The differential currents are amplified using a current mirror with a *W*/*L* ratio of *k*. The amplified currents are converted to voltage signals using *R_1_* and are demodulated to the baseband. The output voltage V*_OUT_* can be expressed as in Equation (1).

*V_OUT_ = 2 × k × R_1_ × I_PD_* (1)

### 2.3. Ambient Light Cancellation (ALC)

**Figure 3:**
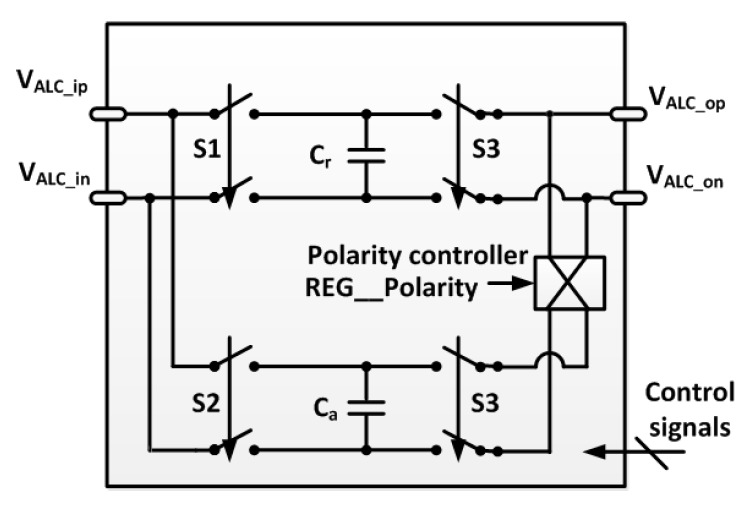
Detailed architecture of the ALC block.

The ambient light component of the detected PPG signal can be removed in the ALC block, which is shown in Figure 3. When the LED light turns on, the measured signal corresponding to both the LED light and the ambient light is sampled at the charge reservoir *C_r_*, and when the LED light turns off, the measured signal corresponding to only the ambient light is sampled at another charge reservoir, *C_a_*. Next, charge redistribution occurs to cancel the ambient light component by reconnecting the two charge reservoirs with reversed polarity.

**Figure 4:**
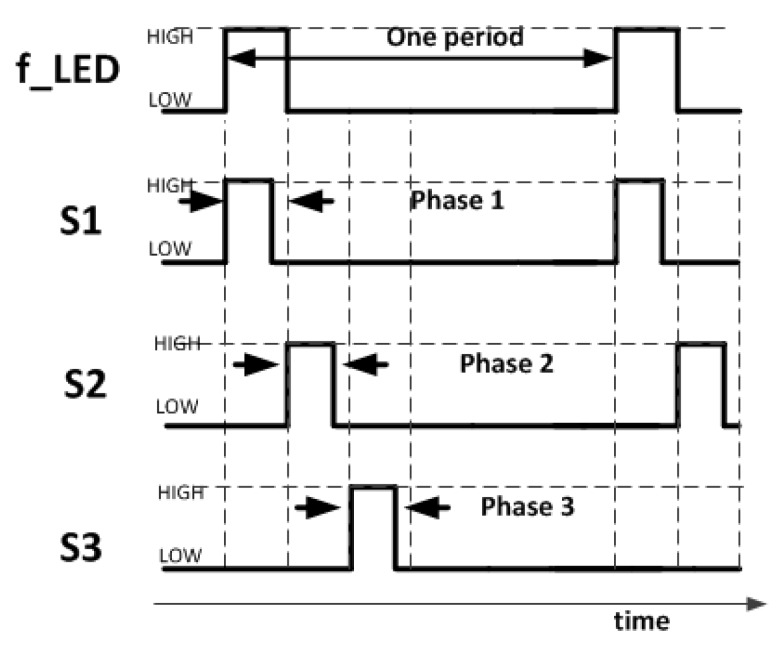
Timing diagram of operation clock for LED power-gating and alternating sampling and charge redistribution.

A detailed diagram of the switch operation clock is shown in Figure 4. In Phase 1, Switch S1 is connected, and Switches S2 and S3 are disconnected. During Phase 1, the differential signal *V_ALC_ipn_ph1_* is sampled in the charge reservoir *C_r_*, and the saved charge can be expressed as follows.

*Q_r_ = C_r_ × V_ALC_ipn_ph1_* (2)

In Phase 2, Switch S2 is connected, and Switches S1 and S3 are disconnected. During Phase 2, the differential signal *V_ALC_ipn_ph2_* is sampled in the charge reservoir *C_a_*, and the saved charge is expressed as follows:

*Q_a_ = C_a_ × V_ALC_ipn_ph2_* (3)

In Phase 3, Switch S3 is connected, and Switches S1 and S2 are disconnected. When the control signal REG__Polarity is in the logic state “high”, the positive and negative nodes of the reservoir *C_r_* are connected to the negative and positive nodes of the reservoir *C_a_*, respectively. In contrast, when the control signal REG__Polarity is in the logic state “low”, the positive and negative nodes of *C_r_* are connected to the positive and negative nodes of *C_a_*, respectively. The polarity controller is used to evaluate the efficacy of the proposed ALC technique.

In this manner, the sampled charges in *C_r_* and *C_a_* are redistributed, and the differential output voltage *V_ALC_opn_* at the ALC block is expressed as follows:

*V_ALC_opn_ = (C_r_ × V_ALC_ipn_ph1_ − C_a_ × V_ALC_ipn_ph2_)/(C_r_ + C_a_)* (4)

When this technique is implemented, sampling charge reservoirs of the same size will be used; therefore, Equation (4) can be simplified to the following:

*V_ALC_opn_ = (V_ALC_ipn_ph1_ − V_ALC_ipn_ph2_)/2* (5)

The sampled signal *V_ALC_ipn_ph1_* in Phase 1 includes the desired signal *V_ALC_ipn_LED_* corresponding to the LED light and the undesired signal *V_ALC_ipn_AL_* corresponding to the ambient light. The signal component from the ambient light should be removed because it is an uncontrollable noise. The sampled signal *V_ALC_ipn_ph2_* in Phase 2 includes only the undesired signal *V_ALC_ipn_AL_* because the LED light is turned off. Finally, at the output node of the ALC block, the differential signal without the ambient light interference is obtained:

*V_ALC_opn_ = V_ALC_ipn_LED_/2* (6)

### 2.4. Automatic Offset Compensation (AOC)

**Figure 5:**
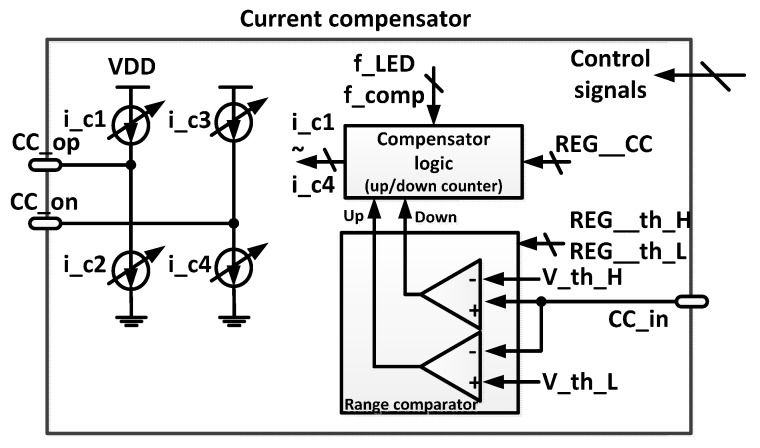
Block diagram of the current compensator block.

The large DC offset current compared with the AC pulse current from the PD is compensated using the current compensator (Figure 5). The range comparator generates information about whether the input signal is oversaturated, undersaturated, or not saturated based on comparison with the upper and lower threshold values, which can be adjusted using the registers REG__th_H and REG__th_L. Depending upon this information, the compensator logic increases or decreases the compensation current. Current compensation is implemented using an updated value at the frequency f_comp and is effective when the LED turns on. The current compensator includes an 8-bit current DAC that compensates a DC offset of up to 30 μA.

### 2.5. Automatic Emitted Light Compensation (AEC)

**Figure 6:**
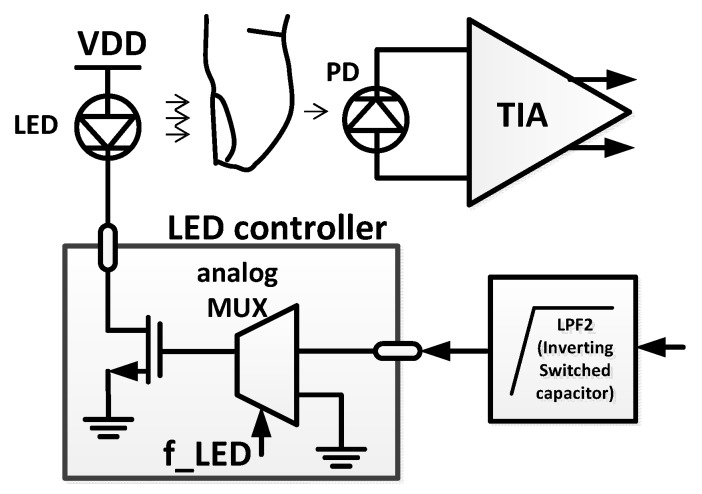
Block diagram of the automatic emitted light compensation (AEC) block.

Most of the DC current is compensated by the current compensator, and the remaining DC current is used for AEC. Figure 6 shows a block diagram of the AEC loop. The detected and amplified signal that passes through the PD and TIA is filtered by an inverting switched-capacitor low-pass filter (ISC-LPF) with a cutoff frequency of 0.1 Hz to extract the DC component. To reduce the average power consumption of the LED, the LED is turned on and off periodically by turning the drain metal–oxide–semiconductor (MOS) on and off. When the LED turns on, the drain MOS is controlled by the filtered signal from the ISC-LPF. For example, when the detected current signal from the PD decreases, the voltage output of the ISC-LPF increases, thus increasing the LED driving current. The increased LED light leads to an increase in the PD current. Therefore, the input signal level of the ADC is maintained.

## 3. Measurement Results and Discussion

**Figure 7:**
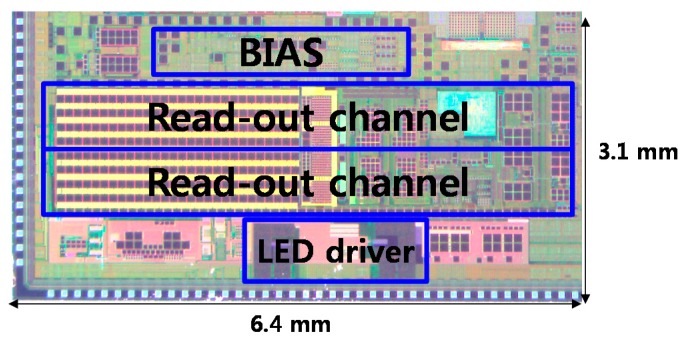
Photograph of chip fabricated by 0.13-μm CMOS process.

**Figure 8:**
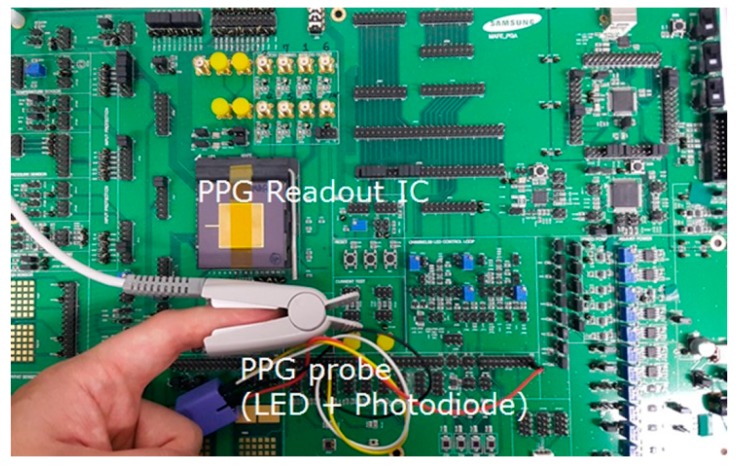
Printed circuit board (PCB) used for evaluation.

**Figure 9:**
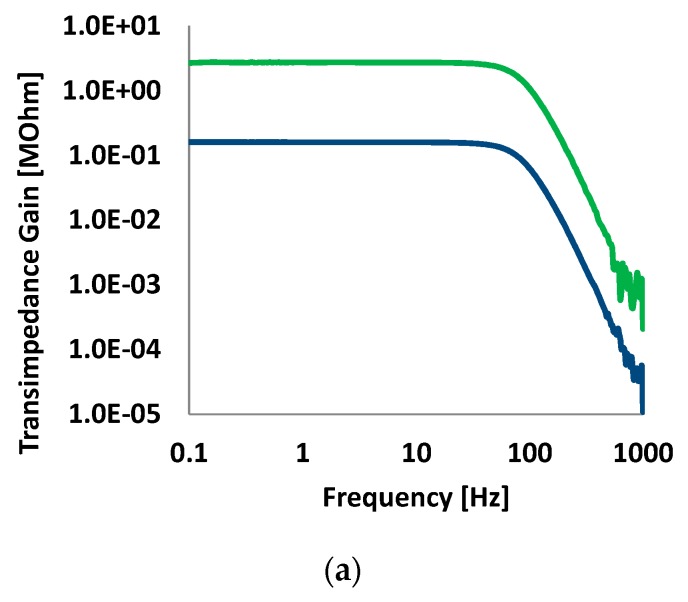

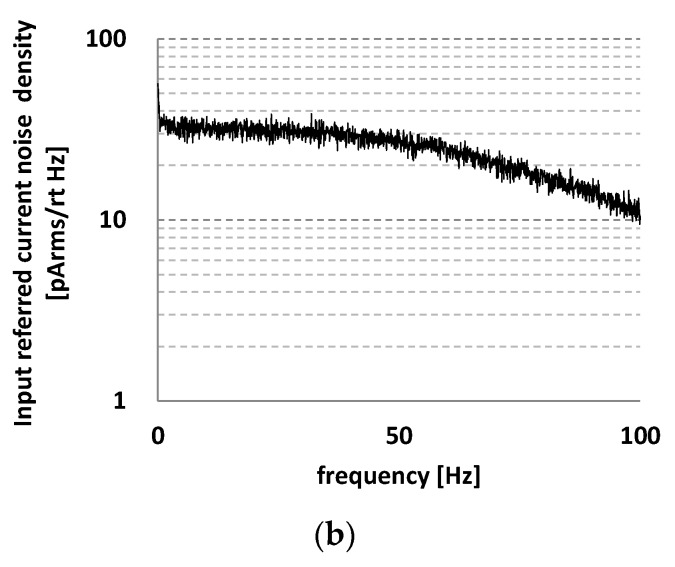
Transimpedance gain and input referred noise. (**a**) Transimpedance gain with minimum and maximum settings; (**b**) Input referred noise spectrum of readout channel.

The designed circuit is implemented using the 0.13-μm complementary metal–oxide–semiconductor (CMOS) process (Figure 7). The overall size, including two readout channels, the LED driver, the bias circuit, and a clock generator is 6.4 mm × 3.1 mm. Figure 8 shows a photograph of the printed circuit board (PCB) used for performance evaluation. The fabricated PPG readout IC and PPG finger probe are mounted on the PCB. Figure 9a shows the frequency response of the readout channel. The readout channel has a transimpedance gain of 160 kOhm to 3.5 MOhm and a low-pass filter with a cutoff frequency of 70 to 500 Hz, which can be set by the register. Figure 9b shows the noise characteristics of the readout channel, which has a noise level of 260 pArms with a bandwidth of 100 Hz. The readout channel, including the TIA, ALC, PGA, and low-pass filters (LPF1 and LPF2) consumes 22 μA at an operation voltage of 1.2 V.

**Figure 10:**
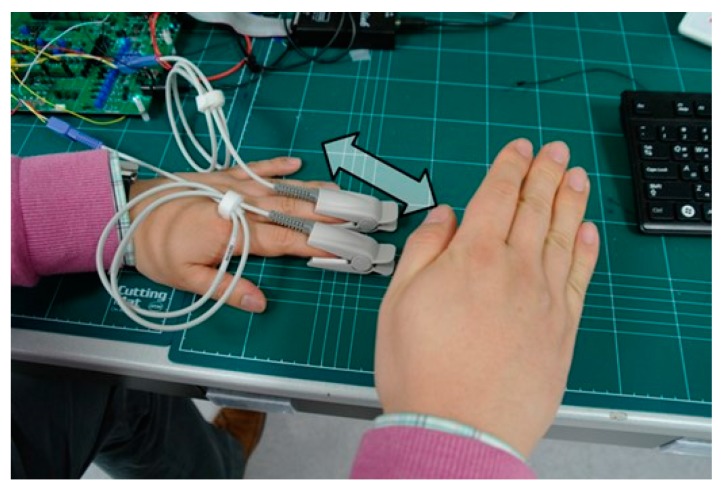
Validation of the ALC method.

**Figure 11:**
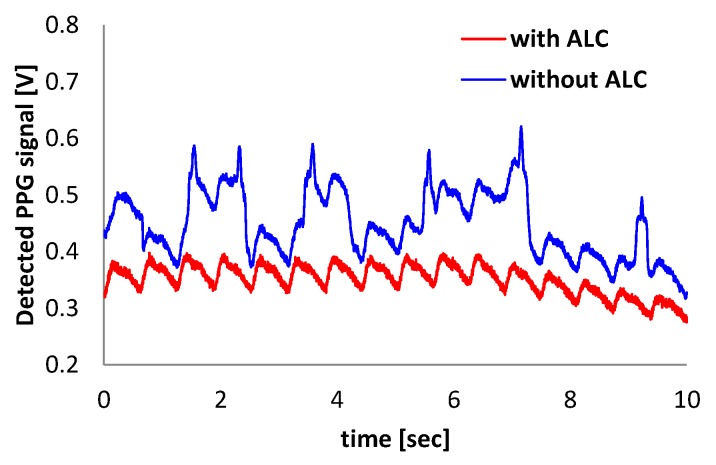
Comparison of output signal with and without the ALC function.

To validate the efficacy of the proposed ALC technique, a PPG (Probe 1) is placed on the index finger, and an another PPG (Probe 2) is placed on the middle finger, as shown in Figure 10. PPG Probes 1 and 2 are connected to readout Channels 1 and 2, respectively. Readout Channel 1 is set to work with the ALC and readout Channel 2 is set not to work with the ALC by changing the connection of a polarity controller, as described in Section 2.2. Owing to the variation of light from a fluorescent lamp, the signal from Channel 2 has a peak component that is influenced by the incident ambient light variation, whereas the signal from Channel 1 is not influenced by the ambient light variation. Figure 11 shows that the proposed alternating sampling and charge redistribution technique can eliminate the ambient light interference.

**Figure 12:**
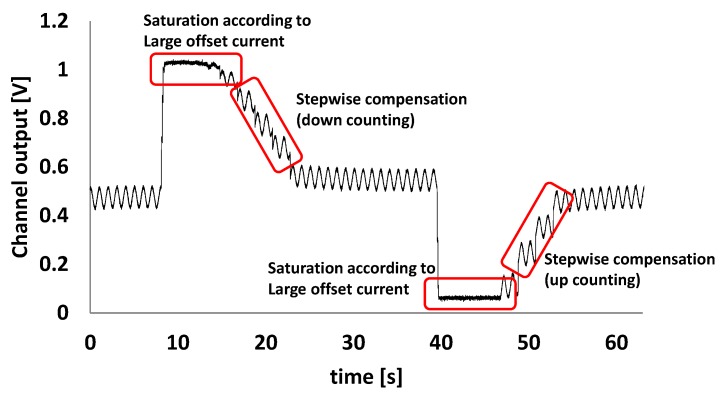
Restoration of saturated signal by the AOC.

To evaluate the ability of the proposed technique to compensate for the large DC offset current, a combined current consisting of a sine wave of 1 Hz and a DC is applied as an input. As shown in Figure 12, when the monitored signal is saturated, the AOC restores the output signal to the desired range. If the monitored signal is higher or lower than the upper or lower threshold voltage of the range comparator in the AOC block, the up/down counting logic and binary weighted current compensator adjust the output offset to the desired value. DC offset compensation of up to 30 μA is achieved experimentally. The stepwise current compensator based on up/down counter logic is operated with a programmable clock frequency (f_comp) of 0.1 Hz to 2 kHz. A subhertz counter operation clock was used intentionally to obtain the results in Figure 12 to show the transient progress of the AOC function. The transient responses of the AOC can be adaptively controlled by programming the clock frequency of the current compensator.

**Figure 13:**
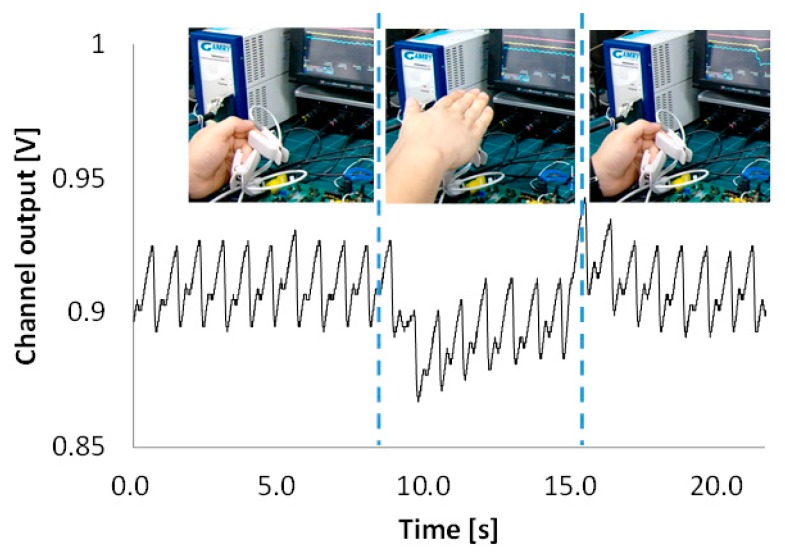
Response of the AEC to artificial interference.

Figure 13 shows the response of the AEC function to external interference. Ambient light is used as the external interference, and the ALC function is turned off. As shown in Figure 13, the ambient light is applied initially. When the ambient light is blocked, the output signal goes down and up for a moment. When the ambient light is applied again, the signal returns gradually to the original level.

**Figure 14:**
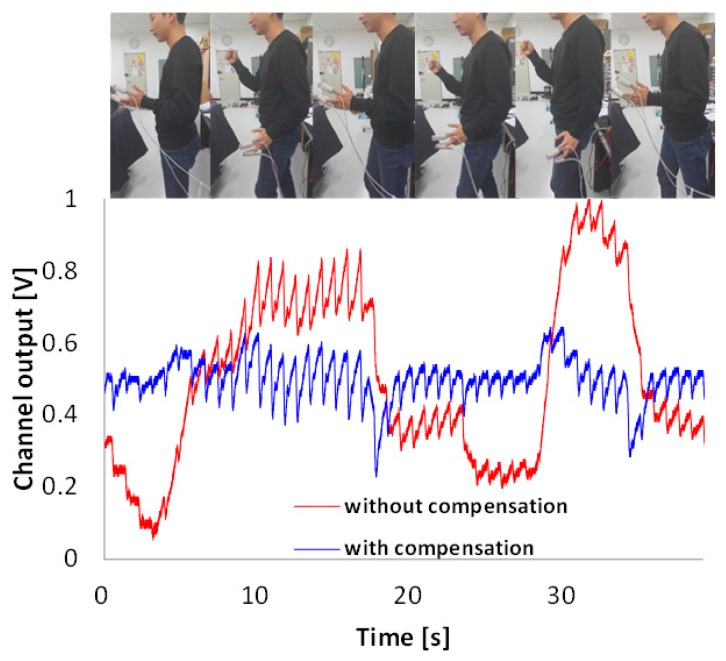
Response of compensation circuit with motion artifact.

Figure 14 shows the measurement results during slow stepping in place with or without the compensation function. Because the fabricated ASIC is embedded on a large evaluation board wired to instruments, the walking experiment was performed using in-place stepping instead of free walking. In the simultaneously acquired signals shown in Figure 14, the output signal without compensation fluctuated severely; however, a relatively stable output signal with compensation appears under motion artifact conditions.

## 4. Conclusions

**Figure 15:**
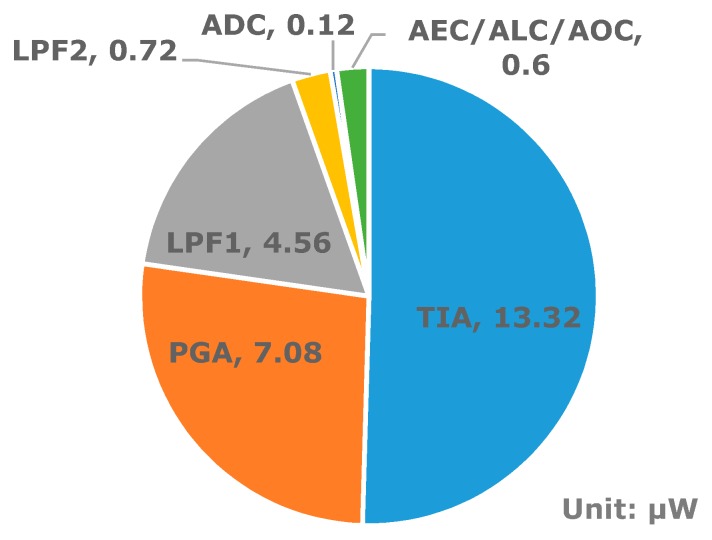
Power breakdown of the presented PPG readout IC.

For a robust PPG application, three compensation methods—An AOC circuit for large DC current offset, ALC through alternating sampling and charge redistribution, and AEC against optical path variation—were implemented, and their efficacy was proved experimentally. To obtain both low-noise and low-power performance, a fully differential TIA with a combined chopper stabilization and switched bias technique was designed. In the ALC circuit, the proposed alternating sampling and charge redistribution technique required only three differential switches and a capacitor. To the best of our knowledge, our proposed ALC technique is the simplest method used to cancel ambient light interference. The transient response of the system can be controlled digitally. Robust PPG acquisition performance with minimized transient response degradation under light interference can be achieved using adaptive digital control from the host microcontroller unit. Performance comparisons to previous works are summarized in Table 1. The power breakdown of the IC is shown in Figure 15. The IC has a low power consumption of 26.4 μW. The additional power consumption for the compensation circuits, *i.e.*, the AEC, ALC, and AOC, is only 0.6 μW. The presented PPG readout IC shows low-power, low-noise, and robust acquisition performance and can be applied to wearable platforms.

**Table 1:** Comparison to previous works, mainly in power consumption and compensation against interference.

	This Work	[11]	[12]	[13]
Technology [μm]	0.13	0.35	1.5	0.18
Readout channel power consumption (w/o LED power) [μW]	26.4	N/A	160	117
Input referred noise [pArms]	260	N/A	N/A	N/A
Automatic offset compensation (AOC)	Yes	Yes	Yes	Yes
Ambient light cancellation (ALC)	Yes	No	No	No
Automatic emitted light compensation (AEC)	Yes	Yes	Yes	No
